# Qualitative changes in clinical records after implementation of pharmacist-led antimicrobial stewardship program: a text mining analysis

**DOI:** 10.1186/s40780-025-00439-0

**Published:** 2025-04-23

**Authors:** Keisuke Sawada, Shuji Kono, Ryo Inose, Yuichi Muraki

**Affiliations:** 1 Department of Pharmacy, Federation of National Public Service Personnel Mutual Aid Associations Hirakata Kohsai Hospital, 1-2-1, Fujisaka-higashi-machi, Hirakata-shi, 573-0153 Osaka Japan; 2 Laboratory of Clinical Pharmacoepidemiology, Kyoto Pharmaceutical University, 5 Misasagi-Nakauchi-cho, Yamashina-ku, Kyoto, 607-8414 Japan

**Keywords:** Antimicrobial stewardship program, Ward pharmacist, Text mining, Co-occurrence network analysis, Medical records, Infectious disease, Medium-sized hospital

## Abstract

**Background:**

Antimicrobial stewardship programs (ASPs) are essential for optimizing antimicrobial use, but many medium-sized hospitals lack infectious disease (ID) specialists. Ward pharmacists can contribute to ASPs, but the qualitative changes in their practice patterns after ASP implementation remains unclear. We aimed to explore the potential of text mining as a novel methodology to evaluate changes in ward pharmacist antimicrobial management practices after ASP implementation in a medium-sized hospital without ID physicians.

**Methods:**

We conducted a retrospective observational analysis of data documented in clinical records by ward pharmacists in a 313-bed community hospital from April 2014 to March 2022. The ASP team conducted weekly reviews of targeted patients, provided feedback to physicians, and shared recommendations with ward pharmacists who then collaborated to optimize antimicrobial therapy. Using Python-based text mining with standardized technical terms and compound word extraction, we performed morphological analysis, co-occurrence network analysis, and hierarchical clustering to evaluate documentation patterns before and after ASP implementation in April 2018. Co-occurrence relationships were assessed using Dice coefficients (threshold, ≥ 0.3), and communities were detected using the Louvain algorithm. Changes in documentation patterns were compared using Fisher’s exact test.

**Results:**

The analysis included 1,353 pre-ASP and 5,155 post-ASP clinical records containing antimicrobial-related terms, which increased from 3.12 to 7.81% of the total pharmacy records. New strong co-occurrence relationships emerged in the post-ASP period for several laboratory parameters (c-reactive protein, 0.646; estimated glomerular filtration rate, 0.594; and white blood cell count, 0.582). Network analysis revealed a shift from medication-focused communities (Medication Review, Prescription Verification, and Patient Education) to infection-focused communities (Infection Assessment, Microbiological Review, and Severe Infection Management). Although Antimicrobial Management was consistently used in both periods (odds ratio [OR]: 0.70, 95% confidence interval [CI]: 0.38–1.20), cross-tabulation analysis increased significantly in Laboratory Monitoring (OR: 1.58, 95% CI: 1.39–1.78) and Infection Assessment (OR: 2.09, 95% CI: 1.85–2.36).

**Conclusions:**

This pilot application of text mining demonstrated potential as a novel methodology for objectively evaluating qualitative changes in clinical practice patterns following ASP implementation, successfully identifying shifts in pharmacists’ documentation focus and providing a foundation for future multi-center validation studies across diverse healthcare settings.

**Supplementary Information:**

The online version contains supplementary material available at 10.1186/s40780-025-00439-0.

## Background

Antimicrobial resistance (AMR) has emerged as a critical global health threat, leading to increased healthcare costs, treatment failure, and mortality rates [1]. The implementation of antimicrobial stewardship programs (ASPs) has been widely recognized as an essential strategy for optimizing antimicrobial use and combating AMR [2]. Guidelines such as those by the Infectious Diseases Society of America emphasize the importance of infectious disease (ID) physicians, along with specialized pharmacists, as core members of ASP teams [3], but the shortage of ID physicians poses significant challenges for healthcare institutions worldwide [4]. This shortage is particularly pronounced in Japan, where ID specialists are unevenly distributed among university hospitals and designated medical institutions [5], with medium-sized community hospitals in particular lacking sufficient resources to implement comprehensive ASPs.

Ward pharmacists focusing on inpatient care make substantial contributions to the optimization of medication therapy across healthcare settings [6]. Their activities encompass a wide range of pharmaceutical care services, including medication order review, drug interaction assessment, medication safety monitoring, and providing medication-related consultation to healthcare professionals [7]. In the context of antimicrobial therapy, some studies have reported the successful involvement of ward pharmacists in specific aspects of antimicrobial stewardship, such as therapeutic drug monitoring-based dose optimization and renal function-based dose adjustment [8, 9]. However, these interventions tend to be limited to specific clinical situations rather than being part of a systematic approach. Comprehensive antimicrobial stewardship activities are still typically conducted by dedicated antimicrobial stewardship pharmacists [10, 11], and evidence regarding the systematic involvement of ward pharmacists in broad antimicrobial stewardship initiatives remains limited, partly due to the challenges in evaluating qualitative changes in clinical practice.

Text mining analysis of medical records has emerged as a valuable tool for evaluating such changes in healthcare practices as well as professional behavior [12]. This approach enables quantitative analysis of qualitative data through techniques such as frequency analysis, co-occurrence network analysis, and cluster analysis [12]. It has been successfully applied in various medical fields, including the analysis of clinical decision-making patterns, assessment of healthcare quality improvements, and evaluation of interprofessional collaboration [13, 14]. However, the application of text mining to evaluate antimicrobial stewardship activities and ward pharmacists’ behavioral changes in antimicrobial management remains unexplored. Therefore, this study aimed to use text mining to analyze the changes in ward pharmacist antimicrobial management practices after the implementation of an ASP.

## Methods

### Aim and study design

This was a single-center retrospective observational study. Its aim was to use Python-based text mining of clinical record text documented by ward pharmacists in a medium-sized Japanese hospital without ID physicians to analyze the qualitative changes in documentation patterns regarding antimicrobial stewardship practices after the implementation of an ASP.

### Setting

In April 2018, we established a pharmacist-led ASP at our hospital, which had no ID physicians. The ASP team comprised nurses (0.1 full-time equivalent [FTE]), clinical microbiologists (0.2 FTE), pharmacists (1.0 FTE), and physicians without ID certification (0.1 FTE). The term “pharmacist-led” in our context refers to the fact that a dedicated ASP pharmacist, who had 3 years of clinical experience when the ASP was initiated in 2018 and consistently served in this role throughout the study period, functioned as the primary coordinator of the ASP activities. In addition to this dedicated ASP pharmacist, a dedicated drug information pharmacist with 15 years of clinical experience who was also a member of the infection control team participated in the weekly conferences. There were no changes in the ASP team membership or significant FTE variations throughout the study period.

The intervention method employed was Prospective Audit and Feedback. The ASP team conducted weekly 2-hour conferences to review patients receiving antipseudomonal antibiotics, anti-methicillin-resistant *Staphylococcus aureus* drugs, prolonged antimicrobial therapy (≥ 14 days), or those with bloodstream infections. Prior to these conferences, the ASP pharmacist was responsible for identifying patients meeting intervention criteria and preparing comprehensive patient summaries. During these conferences, the team discussed the need for adjustments to treatment duration, additional laboratory tests, changes in antimicrobial agents, and optimization of dosing regimens. The ASP recommendations were communicated to attending physicians through electronic medical records and shared with ward pharmacists via email, with direct communication for urgent cases. The ward pharmacists then collaborated with attending physicians to optimize antimicrobial therapy based on these recommendations. Although an Infection Control Team had been active prior to April 2018, these ASP-related activities had not been previously conducted.

### Study data

The study period (April 2014 to March 2022) was divided into two periods using April 2018 (when the ASP was implemented) as the cut-off point—the pre-ASP period (April 2014 to March 2018) and the post-ASP period (April 2018 to March 2022). Clinical records documented by ward pharmacists were extracted from the hospital’s electronic medical record system (provided by Software Service, Inc.). Records containing the term “antimicrobial” (including alternative terms such as “antibiotic,” “antibacterial,” and “anti-infective”) were included for analysis. Records documented by ASP pharmacists were categorized as “Team records” in our electronic medical record system and were excluded. Although no standardized templates were used among pharmacists during the study period, we cannot exclude the possibility that individual pharmacists may have used consistent phrasing in their documentation. We also collected data on the number of ward pharmacists and their years of clinical experience during both periods to assess potential confounding factors.

### Data collection and anonymization

The data extracted from electronic medical records underwent a two-stage anonymization process. First, the medical information management department at Hirakata Kohsai Hospital replaced identifiable information (such as patient identifiers and medical record numbers) with anonymized codes. Second, the researchers transferred this data to a standalone environment and performed morphological analysis and part-of-speech filtering to detect and remove personal identifiers (such as organization names, personal names, and geographical locations) from the free-text portions. The dataset generated after this two-stage anonymization process was stored in a password-protected Microsoft OneDrive. The original data and anonymization correspondence table were securely managed within the hospital.

### Text preprocessing

All analyses were performed using Python 3.9.4. We performed morphological analysis using mecab-python3 (version 1.0.10) with the ipadic dictionary and part-of-speech filtering using the unicodedata2 package (version 16.0.0) on the anonymized dataset. The morphological analysis was executed in the -Ochasen mode for detailed part-of-speech tagging. Parts of speech were classified into nouns, verbs, adjectives, adverbs, interjections, and prenominal adjectives. To extract meaningful words, we excluded certain word categories—suffixes, numbers, pronouns, auxiliary words, and formal nouns from nouns; auxiliary adjectives from adjectives; and all symbols, particles, and auxiliary verbs.

### Standardization of technical terms

Compound terms were extracted using the termextract package (version 0.12b) in Python. Terms were selected based on their importance scores calculated using the log-frequency ratio method (lr_mode = 1, average_rate = True). Words with scores in the top 5% were classified as compound words.

To standardize terminology variations, we used the Medical Subject Headings (MeSH) and Systematized Nomenclature of Medicine (SNOMED) controlled vocabularies. Terms were standardized according to the following procedure: Two pharmacists with dedicated ASP experience collaboratively searched for equivalent MeSH terms for each extracted term using the MeSH Browser (https://meshb.nlm.nih.gov/). When an appropriate MeSH term was found, the extracted term was replaced with the standardized MeSH term. If no suitable MeSH term was available, the pharmacists searched for an equivalent SNOMED term using the SNOMED CT Browser (https://browser.ihtsdotools.org/). Terms that were mapped to the same MeSH or SNOMED identifier (e.g., D000900 and 404684003, respectively) were treated as synonyms to ensure consistent terminology. In cases where the pharmacists encountered difficulty in term mapping, consensus was reached through discussion.

Single alphabet characters and units were excluded as meaningful analysis targets. The dataset with these defined compound words, synonyms, and exclusion words was then reprocessed using morphological analysis to obtain the final processed dataset.

### Co-occurrence analysis

We constructed a co-occurrence network centered on the term “antimicrobial” using the networkx package (version 3.2.1) in Python. As “antimicrobial” appeared in all records and was expected to show large differences in detection frequency with other terms, we used the Dice coefficient to evaluate the strength of co-occurrence relationships. Considering that the Dice coefficient typically yields higher values than the Jaccard coefficient, we set a conservative threshold of ≥ 0.3 to define the presence of co-occurrence relationships. The network was exported in GraphML format and visualized using gephi (version 0.10.1). To ensure network readability, we applied the ForceAtlas layout algorithm, adjusted edge thickness based on the Dice coefficient values, and adjusted node sizes according to their frequency of occurrence. We have depicted only the minimum spanning tree, prioritizing edges with higher coefficients.

Community detection within the co-occurrence network was performed using the Louvain algorithm implemented in the python-louvain package (version 0.16). The best partition was determined using modularity optimization (community.best_partition(G)), which identifies groups of densely connected terms. For the interpretation of the communities, two pharmacists with dedicated ASP experience independently reviewed the terms within each community and assigned names reflecting the primary focus of the grouped terms. Any disagreements were resolved through discussion with a third researcher (a faculty member with experience in hospital infection control). The detailed naming process and term lists for each community are provided in the supplementary materials.

### Cluster analysis

Hierarchical clustering was performed using Ward’s method in the scipy package (version 1.13.1) on terms detected as nodes in the co-occurrence network. Dissimilarity was initially defined as 1- Dice coefficient, but the final cluster distances were computed using Ward’s method, which minimizes the variance increase at each merging step. Based on preliminary examinations of the dendrogram structure, the number of clusters was set to 10% of the total detected terms to balance interpretability. The naming process for the clusters followed the same procedure as described in the co-occurrence analysis section. To evaluate changes in documentation content before and after ASP implementation, we performed cross-tabulation comparing the frequency of occurrence between the pre-ASP and post-ASP periods for the obtained clusters. To exclude the potential impact of the coronavirus disease 2019 (COVID-19) pandemic on our findings, we conducted a sensitivity analysis that excluded data from April 2020 onward (the beginning of the pandemic’s impact in Japan). Odds ratios and their 95% confidence intervals were calculated; statistical comparisons between the periods were conducted using Fisher’s exact test. Statistical significance was set at *p* < 0.05.

### Data management

To ensure reproducibility, the data extraction conditions, and analysis programs were documented in the electronic medical record system. As specified in the data management policy established by the research ethics committee, the original non-anonymized data were designated to be retained at Hirakata Kohsai Hospital for 3 years from the study completion date, and the processed dataset at Kyoto Pharmaceutical University for 5 years. The policy also specified that all data must be destroyed using methods that prevent restoration after these retention periods.

## Results

### Basic characteristics of the text data

The basic characteristics of the study data are presented in Table 1. Ward pharmacist staffing remained relatively stable throughout the study period, with a slight increase in the annual average number after ASP implementation (12.8 to 14.5) and consistent average years of clinical experience (7.3 to 7.4 years). The proportion of records containing the term “antimicrobial” increased substantially, from 1,353 (3.12%) in the pre-ASP period to 5,155 (7.81%) in the post-ASP period. The preprocessing identified 29 compound words, 20 sets of synonyms, and 37 exclusion words (Additional file 1, Supplementary Tables 1 and 2). The morphological analysis resulted in the extraction of 75,148 words (55.5 words per record) in the pre-ASP period and 338,134 words (65.6 words per record) in the post-ASP period.

**Table 1:** Basic characteristics of the study data

	Pre-ASP	Post-ASP
Ward pharmacist staffing		
Number of ward pharmacists at period start	11	13
Annual average number of ward pharmacists	12.8 ± 2.4	14.5 ± 1.3
New pharmacists during the period	9	8
Departing pharmacists during the period	7	6
Average years of clinical experience	7.3 ± 7.8	7.4 ± 5.7
Documentation characteristics		
Total clinical records	43,374	65,982
Records containing “antimicrobial”	1,353 (3.12%)	5,155 (7.81%)
Average characters per record	267.1 ± 116.9	353.1 ± 209.3
Average raw terms per record ^a^	149.1 ± 67.2	197.1 ± 120.5
Average analyzed terms per record ^b^	55.5 ± 24.0	65.6 ± 37.7

Values are presented as mean ± standard deviation where applicable

^a^ Raw terms indicate terms extracted after morphological analysis before filtering

^b^ Analyzed terms indicate terms used for final analysis after morphological analysis, part-of-speech filtering, and standardization

Abbreviations: ASP, antimicrobial stewardship program

### Terms co-occurring with “antimicrobial”

Terms with direct co-occurrence relationships (Dice coefficient ≥ 0.3) with “antimicrobial” were identified—27 terms in the pre-ASP period and 31 terms in the post-ASP period (Table 2). Three terms showed strong co-occurrence relationships (Dice coefficient ≥ 0.5) in both periods—“Laboratory Test” (Dice coefficient: pre-ASP, 0.525; post-ASP, 0.672), “Creatinine” (pre-ASP, 0.507; post-ASP, 0.609), and “Verification” (pre-ASP, 0.681; post-ASP, 0.584). In the pre-ASP period, “Necessity” and “Particularly” were characteristically detected, while in the post-ASP period, new strong relationships emerged with “C-Reactive Protein (CRP)”, “estimated Glomerular Filtration Rate (eGFR)”, “White Blood Cell Count (WBC)”, and “Aspartate Aminotransferase (AST)”.

**Table 2:** Terms with strong co-occurrence relationships with “antimicrobials” (Dice coefficient ≥ 0.3)

	Pre-ASP (*n* = 1,353)		Post-ASP (*n* = 5,155)	
Term	Frequency ^a^	Co-occurrence ^b^	Frequency ^a^	Co-occurrence ^b^
ALT			1,677 (32.5)	0.491
AST			1,740 (33.8)	0.505
Administration	282 (20.8)	0.345	1,378 (26.7)	0.422
Bacterial Test			936 (18.2)	0.307
CRP	338 (25.0)	0.400	2,457 (47.7)	0.646
Caution	420 (31.0)	0.474	1,114 (21.6)	0.355
Ceftriaxone			975 (18.9)	0.318
Continuation			1,041 (20.2)	0.336
Creatinine	459 (33.9)	0.507	2,258 (43.8)	0.609
Decrease			1,056 (20.5)	0.340
Discontinuation			946 (18.4)	0.310
Dosage	296 (21.9)	0.359		
Drugs	428 (31.6)	0.481	1,098 (21.3)	0.351
eGFR	366 (27.1)	0.426	2,179 (42.3)	0.594
Explanation	378 (27.9)	0.437		
Fever			1,044 (20.3)	0.337
Guidance	364 (26.9)	0.424		
Hospitalization			1,014 (19.7)	0.329
Initiation	358 (26.5)	0.419	1,270 (24.6)	0.395
Laboratory Test	482 (35.6)	0.525	2,609 (50.6)	0.672
Management Method	281 (20.8)	0.344		
Med. Adherence	353 (26.1)	0.414		
Med. Reconciliation	310 (22.9)	0.373	916 (17.8)	0.302
Med. Taking	276 (20.4)	0.339	931 (18.1)	0.306
Medicine	370 (27.3)	0.430		
Modification	315 (23.3)	0.378	1,424 (27.6)	0.433
Necessity	618 (45.7)	0.627	1,025 (19.9)	0.332
Neutrophil			1,543 (29.9)	0.461
Oral Administration	327 (24.2)	0.389	1,344 (26.1)	0.414
Particularly	497 (36.7)	0.537		
Prescription	384 (28.4)	0.442	1,258 (24.4)	0.392
Qualitative Test			1,497 (29.0)	0.450
Renal Function	346 (25.6)	0.407		
Report	266 (19.7)	0.329		
SBT/ABPC			1,062 (20.6)	0.342
Safety	434 (32.1)	0.486		
Tablet	387 (28.6)	0.445	1,260 (24.4)	0.393
Take	333 (24.6)	0.395	979 (19.0)	0.319
Tend			1,158 (22.5)	0.367
Verification	698 (51.6)	0.681	2,124 (41.2)	0.584
WBC			2,113 (41.0)	0.582

^a^ Numbers in parentheses indicate the percentage of records containing the corresponding term

^b^ Dice coefficients indicate the strength of co-occurrence relationships

Blank cells indicate that the term was not detected or did not meet the threshold for co-occurrence (Dice coefficient ≥ 0.3) in that period

Abbreviations: ALT, alanine aminotransferase; ASP, antimicrobial stewardship program; AST, aspartate aminotransferase; CRP, C-reactive protein; eGFR, estimated glomerular filtration rate; Med., medication; SBT/ABPC, sulbactam/ampicillin; WBC, white blood cell count

### Network analysis of co-occurring terms

**Fig. 1:**
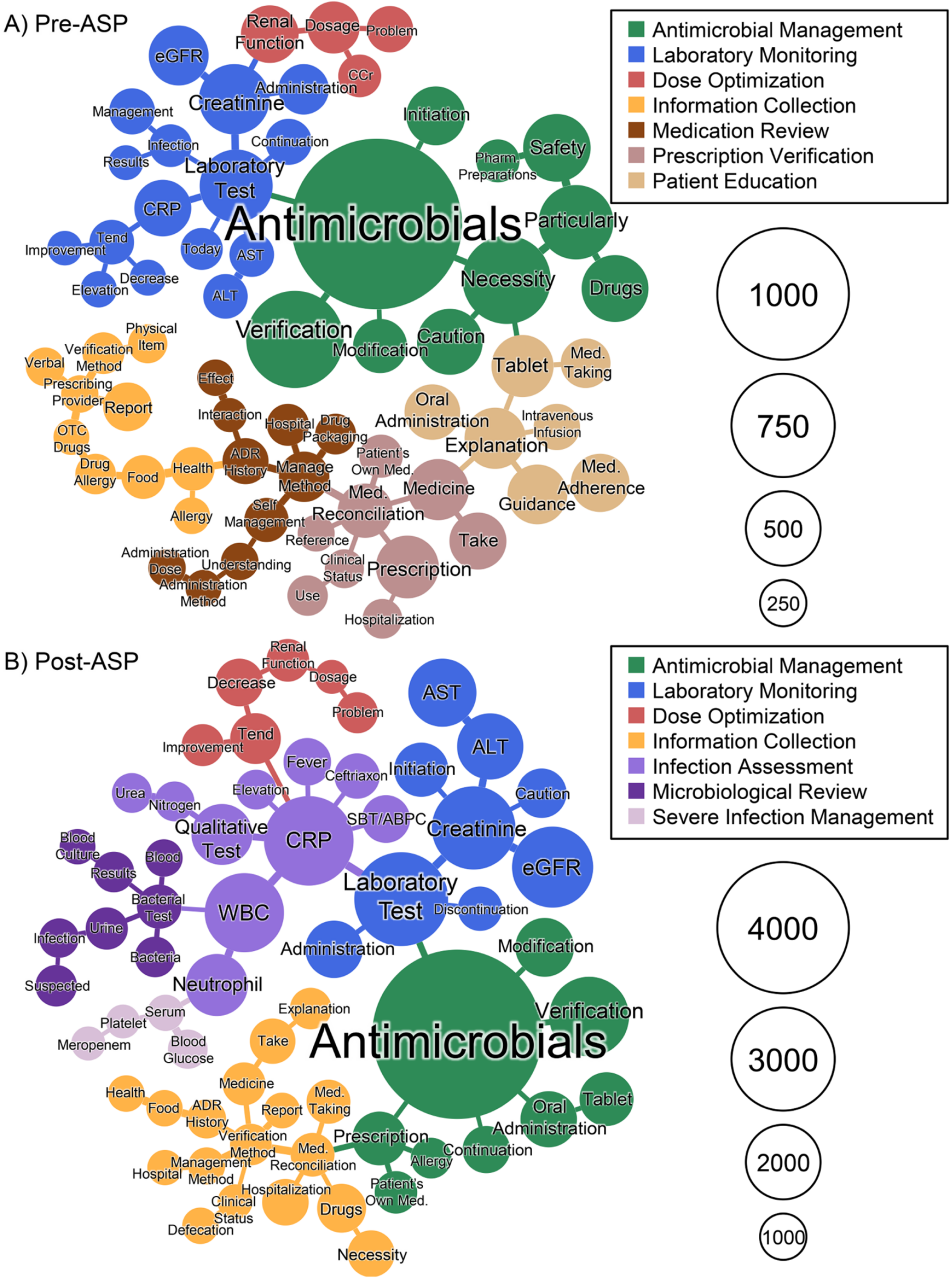
Co-occurrence network analysis of terms related to antimicrobials. Abbreviations: ALT, alanine aminotransferase; AST, aspartate aminotransferase; ASP, antimicrobial stewardship program; CRP, C-reactive protein; eGFR, estimated glomerular filtration rate; Med., medication; SBT/ABPC, sulbactam/ampicillin; WBC, white blood cell count

A total of 84 co-occurring terms formed a network, with 66 nodes in the pre-ASP period and 63 nodes in the post-ASP period (Fig. 1). Louvain algorithm analysis detected seven communities in each period (Additional file 1, Supplementary Table 3). Four communities were common to both periods: Laboratory Monitoring, Antimicrobial Management, Information Collection, and Dose Optimization. The pre-ASP period was characterized by communities related to Medication Review, Prescription Verification, and Patient Education, while the post-ASP period showed newly emerged communities related to Infection Assessment, Microbiological Review, and Severe Infection Management.

### Cluster analysis and cross-tabulation

**Fig. 2:**
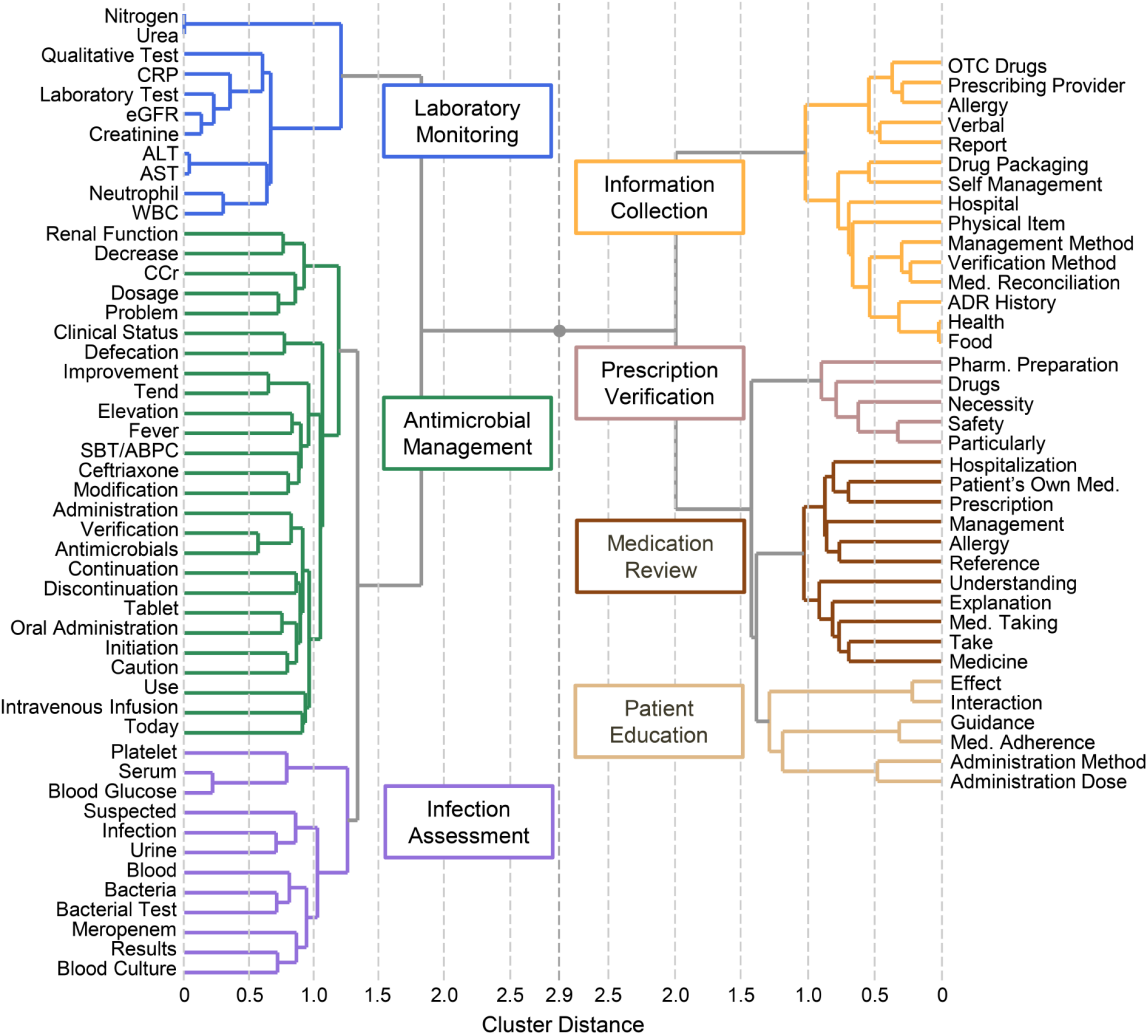
Hierarchical cluster analysis of terms related to antimicrobials. The value 2.9 represents the maximum cluster distance between groups. Abbreviations: ADR, adverse drug reaction; ALT, alanine aminotransferase; AST, aspartate aminotransferase; CCr, creatinine clearance; CRP, C-reactive protein; eGFR, estimated glomerular filtration rate; Med., medication; OTC, over-the-counter; Pharm., pharmaceutical; SBT/ABPC, sulbactam/ampicillin; WBC, white blood cell count

Ward’s method of hierarchical cluster analysis of the 84 terms identified in the network analysis yielded seven groups (Fig. 2). Cross-tabulation analysis comparing the pre- and post-ASP periods showed significant increases in Laboratory Monitoring and Infection Assessment and significant decreases for Prescription Verification, Medication Review, Patient Education, and Information Collection. Only Antimicrobial Management remained consistently high in both periods (Table 3). Sensitivity analysis comparing the pre-ASP period with only the early post-ASP period (April 2018 to March 2020, *n* = 1,400) showed similar patterns to the main analysis, with significant increases in Laboratory Monitoring (OR: 1.79, 95% CI: 1.54–2.09, *p* < 0.01) and Infection Assessment (OR: 2.05, 95% CI: 1.75–2.39, *p* < 0.01) (Additional file 1, Supplementary Table 4).

**Table 3:** Changes in Documentation patterns before and after ASP implementation

	Pre-ASP (*n* = 1,353)	Post-ASP (*n* = 5,155)	Odds ratio (95% CI)	*p* value
Antimicrobial Management	1,337 (98.8)	5,068 (98.3)	0.70 (0.38 − 1.20)	0.22
Laboratory Monitoring	617 (45.6)	2,934 (56.9)	1.58 (1.39 − 1.78)	< 0.01
Infection Assessment	598 (44.2)	3,214 (62.4)	2.09 (1.85 − 2.36)	< 0.01
Information Collection	494 (36.5)	1,405 (27.3)	0.65 (0.57 − 0.74)	< 0.01
Prescription Verification	746 (55.1)	1,896 (36.8)	0.47 (0.42 − 0.54)	< 0.01
Medication Review	992 (73.3)	3,264 (63.3)	0.63 (0.55 − 0.72)	< 0.01
Patient Education	591 (43.7)	917 (17.8)	0.28 (0.24 − 0.32)	< 0.01

Values are expressed as n (%). Statistical comparisons were performed using Fisher’s exact test. p values < 0.05 were considered statistically significant

Abbreviations: ASP, antimicrobial stewardship program; CI, confidence interval

## Discussion

This study used text mining analysis to evaluate changes in ward pharmacist interventions for antimicrobial therapy before and after the implementation of an ASP. The analysis revealed characteristic changes in documentation patterns—specifically, an increase in ID-related terms and a relative decrease in medication guidance-related terms. Network analysis demonstrated a shift from communities related to Medication Review, Prescription Verification, and Patient Education in the pre-ASP period to those focused on Microbiological Review, Infection Assessment, and Severe Infection Management in the post-ASP period.

In the context of antimicrobial therapy, CRP level and WBC are essential indicators for assessing infection severity and treatment efficacy [15]; moreover, renal function and liver function test results (eGFR and AST level, respectively) are crucial for dose adjustment [16, 17], and microbiological test results are essential for appropriate antimicrobial selection and improving treatment outcomes [18, 19]. In our study, new co-occurrence relationships involving these specific laboratory parameters emerged in the post-ASP period, beyond the general association with Laboratory Tests in the pre-ASP period. Additionally, the emergence of the Microbiological Review community suggests that ward pharmacists may have evolved their practices to include more specialized therapeutic support, particularly in terms of pathogen-based antimicrobial selection.

The basic roles of ward pharmacists include medication management activities such as safety verification and providing medication guidance [7, 20, 21]. In our analysis, while terms related to Microbiological Review and Infection Assessment increased after ASP implementation, the proportion of terms related to Medication Review, Patient Education, and Prescription Verification decreased. However, the overall clinical documentation by pharmacists also increased 1.5-fold after ASP implementation, with antimicrobial-related records showing a particularly marked increase (from 3.12 to 7.81% of the total records). These findings suggest that ward pharmacists expanded their expertise into antimicrobial stewardship while maintaining their basic medication management activities.

Previous studies evaluating ASP effectiveness focused primarily on large hospitals with adequate staff, including one to two ID physicians, at least one dedicated pharmacist, and one or more infection control nurses [10, 22, 23]. In contrast, even among legally designated medical institutions for IDs in Japan, only 33.3% have ID specialists [5], and many hospitals struggle to allocate dedicated ASP staff owing to resource constraints [24]. Although several reports have described the feasibility and effectiveness of ASPs in resource-limited settings [25–27], very few studies have examined how ward pharmacists adapt their practices to support antimicrobial stewardship in hospitals without ID specialists. Our text mining analysis provides objective evidence on how ward pharmacists can evolve their practice patterns to support antimicrobial stewardship in resource-limited settings.

Conventional evaluations of ASP activities have primarily focused on quantitative indicators such as antimicrobial use and treatment duration [28]. Although these measures are essential for assessing program effectiveness, they may not fully capture the evolution of ward pharmacist practices and their collaboration with ASP teams. Text mining of medical records has emerged as a complementary analytical approach that can reveal changes in clinical practice patterns [29, 30]. In this study, this analytical method provided unique insights into how ward pharmacists adapt their roles and responsibilities in response to new antimicrobial stewardship initiatives.

Nonetheless, this study had some limitations. First, text mining cannot be used to establish causal relationships, making it impossible to determine whether the changes in documentation patterns resulted from improved pharmacist expertise or ASP interventions. Although our data showed that the average years of clinical experience of ward pharmacists remained relatively stable throughout the study period (pre-ASP 7.3 years vs. post-ASP 7.4 years), we cannot exclude the possibility that changes in experience levels influenced the results. Additionally, while our sensitivity analysis showed similar results when excluding the COVID-19 pandemic period, we cannot rule out the possibility that major events that significantly impact awareness of IDs, such as the pandemic, may have influenced our findings.

Second, as this was a single-center retrospective study, the generalizability of our findings remains limited. However, the primary aim of this study was to qualitatively evaluate antimicrobial stewardship activities using a novel text mining approach. We recognize that this investigation serves as a pilot study proposing methodologies for handling unprocessed electronic medical record data containing personal information in text mining research and for evaluating data after morphological analysis. Future studies applying this methodology across healthcare institutions with diverse backgrounds and examining its applicability to other healthcare professionals would contribute to building more robust evidence.

## Conclusions

As a pilot study, we applied a novel text mining approach to qualitatively evaluate antimicrobial stewardship activities in a single Japanese hospital. Future research applying this methodology across diverse healthcare institutions and examining its applicability to other healthcare professionals will contribute to building more robust evidence for the evolution of pharmacy practice in antimicrobial stewardship.

## Electronic supplementary material

Below is the link to the electronic supplementary material.

Supplementary Material 1

## Data Availability

The datasets generated and/or analyzed during the current study are not publicly available due to privacy concerns and institutional policies regarding medical records but are available from the corresponding author on reasonable request with approval from the Ethics Committees of Hirakata Kohsai Hospital and Kyoto Pharmaceutical University. The detailed protocol for text preprocessing, including the standardized terminology list and compound word definitions, is available in the supplementary materials.

## Abbreviations

ADR: Adverse drug reaction
ALT: Alanine aminotransferase
AMR: Antimicrobial resistance
AST: Aspartate aminotransferase
ASP: Antimicrobial stewardship program
CCr: Creatinine clearance
CI: Confidence interval
COVID-19: Coronavirus disease-2019
CRP: C-reactive protein
eGFR: Estimated glomerular filtration rate
FTE: Full-time equivalent
ID: Infectious disease
Med.: Medication
MeSH: Medical Subject Headings
MRSA: Methicillin-resistant Staphylococcus aureus
OR: Odds ratio
OTC: Over-the-counter
Pharm.: Pharmaceutical
SBT/ABPC: Sulbactam/ampicillin
SNOMED: Systematized Nomenclature of Medicine
WBC: White blood cell count
