# Supplementary material for: Qualitative changes in clinical records after implementation of pharmacist-led antimicrobial stewardship program: a text mining analysis
Source: J Pharm Health Care Sci. 2025 Apr 23;11:34. doi: 10.1186/s40780-025-00439-0 (PMC12020132; doi:10.1186/s40780-025-00439-0)
Supplement: Supplementary file 1 — Supplementary Material 1 [file 40780_2025_439_MOESM1_ESM.docx]

**Supplementary Table 1. Term standardization and synonyms used in the text mining analysis**

| **Detected Term** | **Expression Form** | **Japanese** | **Compound Word ^a^** | **Synonyms** | **MeSH/SNOMED** |
| --- | --- | --- | --- | --- | --- |
| Administration | Administration | 投与 | − | − | MeSH: D000284 |
| Administration Dose | Administration Dose | 用量 | − | − | MeSH: D004302 |
| Administration Method | Administration Method | 用法 | − | − | SNOMED: 422037009 |
| Adverse Effect Monitoring | Adverse Effect Monitoring | 副作用確認 | Yes (114,488) | − | MeSH: D004358 |
| Alanine Transaminase | ALT | ALT | − | GPT, ALT (GPT) | MeSH: D000410 |
| Albumin | Albumin | アルブミン | − | ALB, アルブミン(ALB) | MeSH: D012693 |
| Allergy | Allergy | アレルギー | − | − | MeSH: D000485 |
| Antimicrobial Agents | Antimicrobials | 抗菌薬 | − | 抗菌剤, 抗菌物質, 抗生薬, 抗生剤, 抗生物質 | MeSH: D000900 |
| Aspartate Aminotransferases | AST | AST | − | GOT, AST(GOT) | MeSH: D001219 |
| Aspiration Pneumonia | Aspiration Pneumonia | 誤嚥性肺炎 | Yes (127,079) | − | MeSH: D011014 |
| Bacteria | Bacteria | 菌 | − | − | MeSH: D001419 |
| Bacterial Test | Bacterial Test | 細菌検査 | Yes (841,354) | − | MeSH: D015673 |
| Blood | Blood | 血 | − | − | MeSH: D001769 |
| Blood Culture | Blood Culture | 血液培養 | − | 血培 | MeSH: D001778 |
| Blood Glucose | Blood Glucose | 血糖 | − | − | MeSH: D001786 |
| C-Reactive Protein | CRP | CRP | − | − | MeSH: D002441 |
| Caution | Caution | 注意 | − | − | SNOMED: 225390008 |
| Cefazolin | CEZ | セファゾリン | − | CEZ | MeSH: D002443 |
| Cefmetazole | CMZ | セフメタゾール | − | CMZ | MeSH: D015088 |
| Ceftriaxone | Ceftriaxone | セフトリアキソン | − | CTRX | MeSH: D015033 |
| Clinical Protocols | Clinical Protocols | 状況紹介 | Yes (162,660) | − | MeSH: D016581 |
| Clinical Status | Clinical Status | 状況 | − | − | SNOMED: 246188002 |
| Continuation | Continuation | 継続 | − | − | SNOMED: 255238004 |
| Creatinine | Creatinine | クレアチニン | − | − | MeSH: D003404 |
| Creatinine Clearance | CCr | CCr | − | − | MeSH: D016223 |
| Decrease | Decrease | 低下 | − | − | SNOMED: 255396002 |
| Defecation | Defecation | 排便 | − | − | MeSH: D003671 |
| Discharge prescription | Discharge prescription | 退院処方 | Yes (119,057) | − | SNOMED: 422425002 |
| Discontinuation | Discontinuation | 中止 | − | − | SNOMED: 255594003 |
| Dosage | Dosage | 投与量 | Yes (407,921) | − | MeSH: D004364 |
| Drug Allergy | Drug Allergy | 薬剤アレルギー | Yes (456,178) | − | MeSH: D000640 |
| Drug discontinued | Drug discontinued | 中止薬 | Yes (160,100) | − | SNOMED: 423423001 |
| Drug Packaging | Drug Packaging | ヒート | − | − | MeSH: D004334 |
| Drug-related event | Drug-related event | 薬剤性 | Yes (114,048) | − | SNOMED: 419511003 |
| Drugs | Drugs | 薬剤 | − | − | MeSH: D004341 |
| Effect | Effect | 作用 | − | − | MeSH: D004624 |
| Elevation | Elevation | 上昇 | − | − | SNOMED: 255395003 |
| estimated Glomerular Filtration Rate | eGFR | eGFR | − | − | MeSH: D005919 |
| Explanation | Explanation | 説明 | − | − | SNOMED: 363679005 |
| Fever | Fever | 発熱 | − | − | MeSH: D005334 |
| Follow-up Assessment | Follow-up Assessment | 経過確認 | Yes (563,065) | − | MeSH: D005500 |
| Food | Food | 食品 | − | − | MeSH: D005502 |
| Guidance | Guidance | 指導 | − | − | SNOMED: 443938003 |
| Health | Health | 健康 | − | − | MeSH: D006262 |
| History of Adverse Drug Reaction | ADR History | 副作用歴 | Yes (201,995) | − | SNOMED: 416098002 |
| Hospital | Hospital | 院 | − | − | MeSH: D006761 |
| Hospitalization | Hospitalization | 入院 | − | − | MeSH: D006760 |
| Improvement | Improvement | 改善 | − | − | SNOMED: 385432009 |
| Infection | Infection | 感染 | − | − | MeSH: D007239 |
| Initiation | Initiation | 開始 | − | − | SNOMED: 423943009 |
| Interaction | Interaction | 相互 | − | − | MeSH: D007378 |
| Intravenous Infusion | Intravenous Infusion | 点滴 | − | − | MeSH: D007333 |
| Laboratory Test | Laboratory Test | 臨床検査 | Yes (1,310,493) | − | MeSH: D015996 |
| Levofloxacin | LVFX | レボフロキサシン | − | LVFX | MeSH: D064704 |
| Management | Management | 管理 | − | − | SNOMED: 308489006 |
| Management Method | Management Method | 管理方法 | Yes (463,370) | − | SNOMED: 129433002 |
| Medication Adherence | Med. Adherence | 服薬 | − | − | MeSH: D008506 |
| Medication Information Sheet | Med. Information Sheet | 薬情 | Yes (207,588) | − | SNOMED: 419121009 |
| Medication Reconciliation | Med. Reconciliation | 持参薬確認 | Yes (2,263,132) | − | MeSH: D056207 |
| Medication Taking | Med. Taking | 服用 | − | − | MeSH: D064419 |
| Medicine | Medicine | 薬 | − | − | MeSH: D008687 |
| Meropenem | Meropenem | メロペネム | − | MEPM, メロペン | MeSH: D017405 |
| Metronidazole | MNZ | フラジール | − | MNZ, アネメトロ | MeSH: D008923 |
| Modification | Modification | 変更 | − | − | SNOMED: 281296001 |
| Necessity | Necessity | 必要 | − | − | SNOMED: 272099008 |
| Neutrophil | Neutrophil | Neutr | − | − | MeSH: D009503 |
| Newly Medication | Newly Med. | 開始薬 | Yes (230,943) | − | SNOMED: 416608005 |
| Nitrogen | Nitrogen | 窒素 | − | − | MeSH: D009584 |
| Oral Administration | Oral Administration | 内服 | − | − | MeSH: D005260 |
| Over-the-Counter Drugs | OTC Drugs | 市販薬 | Yes (299,271) | − | MeSH: D000972 |
| Particularly | Particularly | 特に | − | − | SNOMED: 255295009 |
| Patient's Own Medication | Patient’s Own Med. | 持参薬 | Yes (1,540,516) | − | SNOMED: 413477004 |
| Personal Medication Record | Personal Med. Record | 薬手帳 | Yes (1,195,510) | − | MeSH: D008490 |
| Pharmaceutical Preparations | Pharm. Preparations | 医薬品 | − | − | MeSH: D004364 |
| Physical Item | Physical Item | 現物 | − | − | SNOMED: 260787004 |
| Platelet | Platelet | 血小板数 | − | Plt, 血小板数(Plt) | MeSH: D010975 |
| Prescribing provider | Prescribing provider | 処方もと | Yes (310,751) | − | SNOMED: 405623001 |
| Prescription | Prescription | 処方 | − | − | MeSH: D011337 |
| Prescription Drug | Prescription Drug | 処方薬 | Yes (181,561) | − | MeSH: D011251 |
| Problem | Problem | 問題 | − | − | SNOMED: 55607006 |
| Procalcitonin | Procalcitonin | プロカルシトニン | − | PCT, プロカルシトニン(PCT) | MeSH: D002117 |
| Qualitative Test | Qualitative Test | 定性 | − | − | MeSH: D011787 |
| Reference | Reference | 参照 | − | − | SNOMED: 370834003 |
| Renal Function | Renal Function | 腎機能 | Yes (564,372) | − | MeSH: D012008 |
| Renal Insufficiency | Renal Insufficiency | 腎機能低下 | Yes (130,845) | − | MeSH: D051437 |
| Report | Report | 報告 | − | − | SNOMED: 371524004 |
| Results | Results | 結果 | − | − | SNOMED: 441742003 |
| Risk | Risk | 可能性 | Yes (163,156) | − | MeSH: D012306 |
| Safety | Safety | 安全 | − | − | MeSH: D012441 |
| Safety Management | Safety Management | 安全管理 | Yes (169,656) | − | MeSH: D012439 |
| Self Management | Self Management | 自己管理 | Yes (339,279) | − | MeSH: D012648 |
| Serum | Serum | 血清 | − | − | MeSH: D012670 |
| Sputum Culture | Sputum Culture | 喀痰培養 | − | 痰培 | MeSH: D013181 |
| Sulbactam/Ampicillin | SBT/ABPC | スルバシリン | − | SBT/ABPC, ABPC/SBT | MeSH: D000667 |
| Sulfamethoxazole-Trimethoprim Combination | SMX/TMP | バクトラミン | − | ST, バクタ | MeSH: D014295 |
| Suspected | Suspected | 疑い | − | − | SNOMED: 415684004 |
| Tablet | Tablet | 錠 | − | − | MeSH: D013600 |
| Take | Take | 飲む | − | − | SNOMED: 417950001 |
| Tazobactam/Piperacillin | TAZ/PIPC | タゾピぺ | − | TAZ/PIPC, PIPC/TAZ | MeSH: D053888 |
| Tend | Tend | 傾向 | − | − | SNOMED: 410666004 |
| Today | Today | 本日 | − | − | SNOMED: 410512000 |
| Understanding | Understanding | 理解 | − | − | SNOMED: 423875000 |
| Urea | Urea | 尿素 | − | − | MeSH: D014508 |
| Uric Acid | Uric Acid | 尿酸 | Yes (161,069) | − | MeSH: D014527 |
| Urine | Urine | 尿 | − | − | MeSH: D014571 |
| Urine Culture | Urine Culture | 尿培養 | − | 尿培 | MeSH: D014571 |
| Use | Use | 使用 | − | − | SNOMED: 360741001 |
| Vancomycin | VCM | バンコマイシン | − | VCM | MeSH: D014640 |
| Verbal | Verbal | 口頭 | − | − | SNOMED: 278435005 |
| Verification | Verification | 確認 | − | − | SNOMED: 398166005 |
| Verification Method | Verification Method | 確認方法 | Yes (620,965) | − | SNOMED: 386053000 |
| Watchful Waiting | Watchful Waiting | 経過観察 | Yes (138,002) | − | MeSH: D057885 |
| White Blood Cell Count | WBC | 白血球数 | − | WBC, 白血球数(WBC) | MeSH: D005118 |

^a^ "Yes" indicates that the term was classified as a compound word based on the importance score calculated using the log-frequency ratio (LR) method. The score is shown in parentheses, with higher values indicating greater importance. "-" indicates that the term was not classified as a compound word.

^b^ This column lists alternative terms or abbreviations that were treated as synonyms for the detected term. "-" indicates that no synonyms were identified for the corresponding term.

Terms were standardized according to Medical Subject Headings (MeSH) and Systematized Nomenclature of Medicine (SNOMED) terminology. Japanese terms and their variations were mapped to standardized English terms to ensure consistency in the text mining analysis. Abbreviations and alternative expressions commonly used in clinical practice were included as synonyms.

**Supplementary Table 2. Terms excluded from the text mining analysis**

| Units | Mg, dL, ML, mmol, g, mg, Kg, min, mEq, Ng, NA |
| --- | --- |
| Single Alphabets | A, B, C, D, E, F, G, H, I, J, K, L, M, N, O, P, Q, R, S, T, U, V, W, X, Y, Z |

These terms were excluded from the morphological analysis to avoid inappropriate word segmentation and ensure meaningful text mining results. Units were excluded as they do not contribute to the semantic analysis of antimicrobial stewardship activities. Single alphabets were excluded as they typically represent abbreviated terms or variables that could introduce noise into the analysis.

**Supplementary Table 3. Communities and clusters identified in the text mining analysis**

**A. Communities detected in the pre-ASP period**

| **Associated Terms** | **Clinical Expert 1** | **Clinical Expert 2** | **Clinical Expert 3** |
| --- | --- | --- | --- |
| Laboratory Test, Creatinine, eGFR, CRP, Administration, Continuation, Management, AST, ALT, Tend, Infection, Today, Decrease, Elevation, Results, Improvement | Laboratory Monitoring | Evaluation of Laboratory Results | Laboratory Monitoring |
| Management Method, ADR History, Self Management, Hospital, PTP Sheet, Administration Dose, Understanding, Effect, Administration Method, Interaction | Medication Review | Assessment of Medication Adherence | Medication Review |
| Report, Food, Health, Verification Method, Drug Allergy, Physical Item, Verbal, Prescribing Provider, Allergy, OTC Drugs | Information Collection | Collection of Patient Information | Information Collection |
| Antimicrobials, Verification, Necessity, Particularly, Safety, Verification, Drugs, Caution, Initiation, Modification, Pharm. Preparation | Antimicrobial Management | Management of Antimicrobial Use | Antimicrobial Management |
| Prescription, Medicine, Take, Med. Reconciliation, Patient's Own Med., Use, Hospitalization, Clinical Status, Reference | Prescription Verification | Regular Medication Review | Prescription Verification |
| Tablet, Explanation, Guidance, Med. Adherence, Oral Administration, Med. Taking, Intravenous Infusion | Patient Education | Patient Counseling | Patient Education |
| Renal Function, Dosage, Problem, CCr | Dose Optimization | Renal Function Assessment | Dose Optimization |

**B. Communities detected in the post-ASP period**

| **Associated Terms** | **Clinical Expert 1** | **Clinical Expert 2** | **Clinical Expert 3** |
| --- | --- | --- | --- |
| Drugs, Necessity, Hospitalization, Take, Med. Taking, Med. Reconciliation, Medicine, Verification Method, Explanation, ADR History, Management Method, Health, Defecation, Food, Report, Hospital, Clinical Status | Information Collection | Collection of Patient Information | Information Collection |
| CRP, WBC, Neutrophil, Qualitative Test, SBT/ABPC, Fever, Ceftriaxone, Elevation, Nitrogen, Urea | Infection Assessment | Evaluation of Infection Treatment | Infection Assessment |
| Antimicrobials, Verification, Modification, Oral Administration, Tablet, Prescription, Continuation, Patient's Own Med., Allergy | Antimicrobial Management | Management of Antimicrobial Use | Antimicrobial Management |
| Bacterial Test, Urine, Suspected, Results, Bacteria, Infection, Blood, Blood Culture | Microbiological Testing | Microbiology Result Review | Microbiological Review |
| Laboratory Test, Creatinine, eGFR, AST, ALT, Administration, Initiation, Caution, Discontinuation | Laboratory Monitoring | Evaluation of Laboratory Results | Laboratory Monitoring |
| Tend, Decrease, Renal Function, Improvement, Problem, Dosage | Dose Optimization | Renal Function Assessment | Dose Optimization |
| Blood Glucose, Serum, Meropenem, Platelet | Additional Monitoring | Management of Severe Infections | Severe Infection Management |

**C. Hierarchical clusters**

| **Key Terms** | **Clinical Expert 1** | **Clinical Expert 2** | **Clinical Expert 3** |
| --- | --- | --- | --- |
| Nitrogen, Urea, Qualitative Test, CRP, Laboratory Test, eGFR, Creatinine, ALT, AST, Neutrophil, WBC | Laboratory Monitoring | Evaluation of Laboratory Results | Laboratory Monitoring |
| Renal Function, Decrease, CCr, Dosage, Problem, Clinical Status, Defecation, Improvement, Tend, Elevation, Fever, SBT/ABPC, Ceftriaxone, Modification, Administration, Verification, Antimicrobials, Continuation, Discontinuation, Tablet, Oral Administration, Initiation, Caution, Use, Intravenous Infusion, Today | Antimicrobial Management | Management of Antimicrobial Use | Antimicrobial Management |
| Platelet, Serum, Blood Glucose, Suspected, Infection, Urine, Blood, Bacteria, Bacterial Test, Meropenem, Results, Blood Culture | Infection Assessment | Evaluation of Infection Treatment | Infection Assessment |
| OTC Drugs, Prescribing Provider, Allergy, Verbal, Report, Drug Packaging, Self Management, Hospital, Physical Item, Management Method, Verification Method, Med. Reconciliation, ADR History, Health, Food | Information Collection | Collection of Patient Information | Information Collection |
| Pharm. Preparation, Drugs, Necessity, Safety, Particularly | Prescription Verification | Regular Medication Review | Prescription Verification |
| Hospitalization, Patient's Own Med., Prescription, Management, Allergy, Reference, Understanding, Explanation, Med. Taking, Take, Medicine | Medication Review | Assessment of Medication Adherence | Medication Review |
| Effect, Interaction, Guidance, Med. Adherence, Administration Method, Administration Dose | Patient Education | Patient Counseling | Patient Education |

Communities were detected using the Louvain algorithm for network analysis, while clusters were identified through hierarchical clustering using Ward's method. Descriptions for each group were determined through independent review by three clinical experts.

**Supplementary Table 4. Changes in documentation patterns before and after ASP implementation: Sensitivity analysis excluding the COVID-19 pandemic period**

|  | Pre-ASP (n=1,353) | early Post-ASP (n=1,400) | Odds ratio (95% CI) | p value |
| --- | --- | --- | --- | --- |
| Antimicrobial Management | 1,337 (98.8) | 1,381 (98.6) | 0.87 (0.42−1.79) | 0.74 |
| Laboratory Monitoring | 617 (45.6) | 841 (60.1) | 1.79 (1.54−2.09) | <0.01 |
| Infection Assessment | 598 (44.2) | 866 (61.9) | 2.05 (1.75−2.39) | <0.01 |
| Information Collection | 494 (36.5) | 480 (34.3) | 0.90 (0.77−1.06) | 0.23 |
| Prescription Verification | 746 (55.1) | 653 (46.6) | 0.71 (0.61−0.83) | <0.01 |
| Medication Review | 992 (73.3) | 908 (64.9) | 0.67 (0.57−0.79) | <0.01 |
| Patient Education | 591 (43.7) | 391 (27.9) | 0.50 (0.42−0.59) | <0.01 |

This sensitivity analysis compares documentation patterns between the pre-ASP period and the early post-ASP period (April 2018 to March 2020), excluding the potential confounding effects of the COVID-19 pandemic.

Values are expressed as n (%). Statistical comparisons were performed using Fisher's exact test. p values <0.05 were considered statistically significant.

Abbreviations: ASP, antimicrobial stewardship program; CI, confidence interval
